# Late glacial (17,060–13,400 cal yr BP) sedimentary and paleoenvironmental evolution of the Sekhokong Range (Drakensberg), southern Africa

**DOI:** 10.1371/journal.pone.0246821

**Published:** 2021-03-17

**Authors:** Malin E. Kylander, Mikaela Holm, Jennifer Fitchett, Stefan Grab, Antonio Martinez Cortizas, Elin Norström, Richard Bindler

**Affiliations:** 1 Department of Geological Sciences and the Bolin Centre for Climate Research, Stockholm University, Stockholm, Sweden; 2 School of Geography, Archaeology and Environmental Studies, University of the Witwatersrand, Johannesburg, South Africa; 3 Facultade de Bioloxía, EcoPast (GI-1553), Universidad de Santiago de Compostela, Santiago de Compostela, Spain; 4 Department of Physical Geography and the Bolin Centre for Climate Research, Stockholm University, Stockholm, Sweden; 5 Department of Ecology and Environmental Sciences, Umeå University, Umeå, Sweden; Centre National de la Recherche Scientifique, FRANCE

## Abstract

Southern Africa sits at the junction of tropical and temperate systems, leading to the formation of seasonal precipitation zones. Understanding late Quaternary paleoclimatic change in this vulnerable region is hampered by a lack of available, reliably-dated records. Here we present a sequence from a well-stratified sedimentary infill occupying a lower slope basin which covers 17,060 to 13,400 cal yr BP with the aim to reconstruct paleoclimatic variability in the high Drakensberg during the Late Glacial. We use a combination of pollen, total organic carbon and nitrogen, δ^13^C, Fourier transform infrared spectroscopy attenuated total reflectance (FTIR-ATR) spectral and elemental data on contiguous samples with high temporal resolution (10 to 80 years per sample). Our data support a relatively humid environment with considerable cold season precipitation during what might have been the final stage of niche-glaciation on the adjoining southern aspects around 17,000 cal yr BP. Then, after an initial warmer and drier period starting ~15,600 cal yr BP, we identify a return to colder and drier conditions with more winter precipitation starting ~14,380 cal yr BP, which represents the first local evidence for the Antarctic Cold Reversal (ACR) in this region. On decadal to centennial timescales, the Late Glacial period was one marked by considerable climatic fluctuation and bi-directional environmental change, which has not been identified in previous studies for this region. Our study shows complex changes in both moisture and thermal conditions providing a more nuanced picture of the Late Glacial for the high Drakensburg.

## Introduction

**Fig 1:**
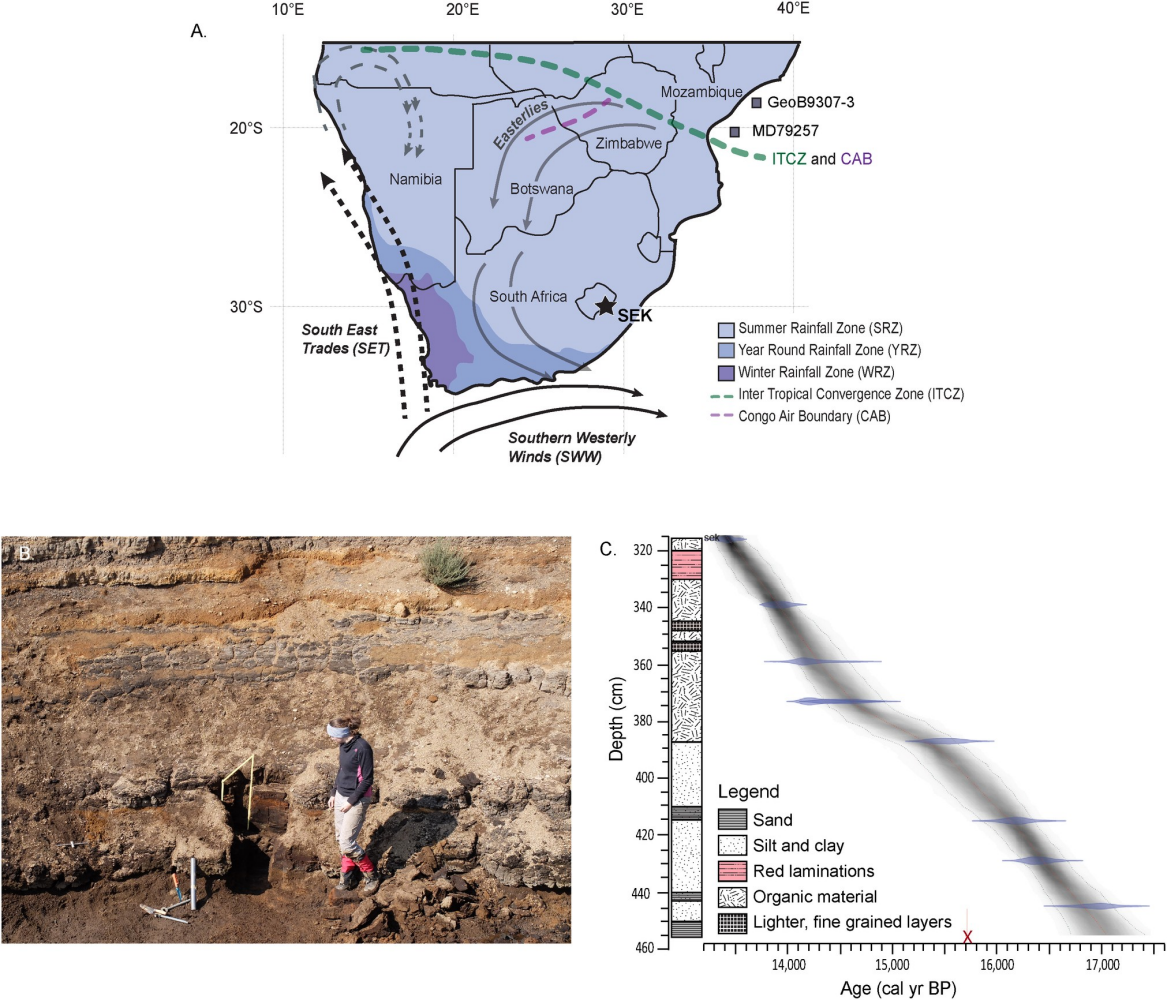
(A) The SEK2016 site is located in eastern Lesotho at ~2900 m a.s.l. As a result of the interplay between tropical and temperate systems southern Africa has zones of seasonally dominant rain during the winter (WRZ, winter rainfall zone) and summer (SRZ, summer rainfall zone). A region also experiences rainfall year-round (YRZ) (coastline and country borders from [14]; rainfall zones redrawn from [1]). Major atmospheric systems are shown here including the Intertropical Convergence Zone (ITCZ), the Congo Air Boundary (CAB), the Southern Westerly Winds (SWW), the South East Trades (SET) and the easterlies (see text for details). Two comparison marine records, GeoB9307-3 and MD79257, are indicated [15,16]. (B) The site presents 5 m thick sediment deposit that has been eroded and exposed. SEK2016 was sampled at the base of this gully. (C) The chronology is based on nine ^14^C dates, one of which was removed from the age model as an outlier.

Southern Africa sits at the interface of tropical and temperate systems, generating a dynamic climate and a heterogeneous landscape. One of the main features of this climatic setting are the seasonally controlled winter and summer rainfall zones (WRZ and SRZ, respectively) [1,2] (Fig 1A). The spatial extent and precipitation intensity of these rainfall zones is linked to changes in global climate systems. Regional analyses indicate a shift in rainfall seasonality over southern Africa in recent decades, and the intense local drought over the Cape region from 2015 to 2017 was linked to the poleward displacement of the southern westerly winds (SWW) [3]. In contrast, the opposite situation has been observed on longer, geological time scales. During the Last Glacial Maximum (LGM), when mountain glaciers reached their maximum ice extent in the mid-latitude southern hemisphere (23,000 and 19,000 cal yr BP) [4–6], it is suggested that an equatorward shift of the SWW extended the WRZ, generating humid conditions over much of southern Africa [e.g., 7–11]. However, a number of open questions related to climate development from the LGM to present still exist. This is in part because high-resolution, continuous, long-term environmental reconstructions are rare and available records are unevenly distributed geographically [12]. While an increasing number of paleorecords from southern Africa are available for the Holocene, the LGM and the Late Glacial (or Last Glacial Transition, ~18,000 to 11,700 cal yr BP [13]) are less frequently represented.

In terms of paleoclimatic studies, the Drakensberg-Maloti region was, until recently, an understudied region of southern Africa, with its rugged and remote topography being difficult to access [17,18]. In keeping with sites elsewhere in southern Africa, available paleo-geomorphological [19–21], archaeological [22,23] and paleoarchive reconstructions [24] suggest a cold and wet LGM in the region. Emerging research on the Late Glacial and the Holocene, however, reveals considerable spatial diversity in climatic and environmental changes, driven by complex interactions between orography, the shifting position and intensity of the SWW and the role of lapse rates in controlling the position of the tree line [17,24–26]. For the Late Glacial, this complexity is exacerbated by the paucity of records available and their low resolution, resulting in a fragmented understanding of this period.

The Late Glacial in southern Africa is of interest when charting the global impact of the Hienrich Stadial 1 (HS1, 17.5 to 14.7 ka [27] and the Antarctic Cold Reversal (ACR, ~14,700 and 13,000 years ago) [28]. Defining the spatial expression of these two events, rooted in the Northern and Southern Hemispheres, respectively, provides important information for decoding the mechanisms of inter-hemispheric coupling. Evidence of the ACR in southern African paleoclimate records is varied. For example, marine sites on the west coast show weak ACR signals [29] while those along the eastern coast show unclear signals [15]. A number of terrestrial sites report a cold and dry period that aligns with the ACR but this is, for example, more obvious in the southern and central, rather than in the northern reaches, of the SRZ [e.g., 9,30,31]. In general, paleoclimate reconstructions from sites below 20°S seem to link more clearly with events in the North Atlantic while those above 40°S follow events in Antarctica [28].

The Lesotho highlands experience relatively high precipitation and low evaporation rates and consequently, the area supports a variety of organic-rich deposits with the potential to capture signals of these key Late Glacial events. One such deposit is the well-stratified sedimentary infill found in a lower slope basin on the north-facing aspect of the Sekhokong Range, Drakensberg (Fig 1A). The site, located at 29°S, was initially investigated by Marker and Whittington [32] and Marker [33,34], who provided a coarse resolution Holocene paleoclimatic history for the region based on descriptive assessments of the sediments [33,34]. More recently, Fitchett et al. [35] re-examined the sedimentary sequence at the site (referred to here as SEK2014; note that it is not the exact same gully sidewall locality as that studied by Marker and Whittington [32]) using sediment characteristics, fossil pollen and diatom assemblages. Results from SEK2014, which spans the last ~16,000 cal yr BP, indicate a relatively dynamic Late Glacial, but this period was only represented by ten discontinuous samples, leaving temporal gaps in our understanding [35]. To address this problem the site was resampled in 2016, recovering the SEK2016 sequence, which spans from ~17,060 to 13,400 cal yr BP. Each contiguous sample represents on average 35 years (range: 10–80 years), providing higher temporal resolution and Late Glacial coverage than previous work. In addition to pollen analyses performed on the older parts of SEK2016, we also acquired total organic carbon (TOC), total nitrogen (TN), stable carbon isotopes (δ^13^C), Fourier transform infrared spectroscopy attenuated total reflectance (FTIR-ATR) spectral data and X-ray fluorescence (XRF) elemental data. Both the spectral and elemental datasets were treated separately using Principal Component Analysis (PCA). In several instances the extracted components from the elemental PCA were represented, and indeed clarified, by the spectral PCA, providing two-fold support for our interpretations. Given the latitude and altitude of our site in the high Drakensberg, we hypothesise that the ACR is expressed as a cold and dry period in SEK2016, as reflected by changes in catchment productivity, redox conditions, sediment source and grain size and vegetation.

## Materials and methods

### Environmental context and study area

Southern Africa is positioned in the transitional zone between tropical and temperate systems. During austral summer (October to March), continental heating brings moisture from the Indian Ocean and the tropical Atlantic Ocean, which is associated with the southward shift of the Inter Tropical Convergence Zone (ITCZ) (Fig 1A). During austral winter (April to September), temperate systems move northwards. At this time the SWW and the South East Trades (SET) bring moisture in from the southwest. The complex interplay between these systems results in southwestern and western coast regions receiving the majority of their rain during winter (WRZ), while central, northern and eastern regions of southern Africa receive the majority of their rain during summer (SRZ). Between these two zones, rain falls year-round (YRZ) [1,2].

The study site is located on the northern aspect of the Sekhokong Range in the high Drakensberg (29°36’31”S, 29°15’54”E) at ~2900 m a.s.l. (Fig 1A). The sedimentary deposit is located in an 800 m wide by 1200 m deep valley head, which is one of three distinct valley heads along the Sekhokong Range. These north-facing valley heads have previously been described as ‘nivation cirques’ [32] and ‘cirque-like hollows’ [36,37]. Their glacial and nival origin has been argued and are instead are proposed to be a product of lithology (high joint density) and enhanced ground seepage controlled weathering and mass transfer [38]. Where the concave slope profiles rapidly flatten to form hollow/cirque floors, the topography is suitable for fan-shaped sedimentation given the rapid loss of transport energy. In that the site has been geologically stable throughout the Quaternary, sedimentation in a relatively undisturbed, layered manner has occurred here since at least the Late Glacial. A perennial stream has eroded through the deposit forming a ~5 m deep gully which exposes alternating layers of organic rich silt and clay, peat, thin sand layers and gravel layers (Fig 1B). Uninterrupted horizontally alternating peat layers are visible along the sediment profile, suggesting an absence of paleo gullies and paleo channels at this site and uniform sedimentation where channelized flow was minimal or absent.

At present, mean seasonal temperatures in the Lesotho Highlands (measured at 3100 m a.s.l.) range from 11°C in January (mid-summer) to 0°C in July (mid-winter) [39]. Since Lesotho is located within the contemporary SRZ, most (>80%) annual precipitation falls during the warmer months (October to April) [40], typically due to thunderstorms and topographically induced instability showers [41]. Mean contemporary annual precipitation at Sekhokong is estimated (in the absence of sufficient long-term rainfall records) at ~1000 mm given its close proximity to the Great Escarpment where orographic uplift enhances rainfall quantity [41]. Contemporary winters are relatively dry and cold, with ~11 snow events per year [42]. The Sekhokong Range is located above the treeline where tussock/meadow grasses, sedges and dwarf shrubs dominate. Our site is situated above the transition zone (2100 to 2700 m a.s.l) where dominant C_4_ grasslands gradually shift to C_3_ grasses that are more tolerant to low temperatures [43,44].

The local geology is comprised of the rather homogenous Sani Pass Basalts, which are estimated to between 193 ± 3 Ma [45] and 182 ± 2 Ma [46] in age. These basalts have been divided into units of various depths including (from the base upwards): the Giant’s Cup (14 m), Agate Vale (70 m), Sakeng (84 m), Mkhomazana (6 m) and Lesotho (625 m) Units. Tuff layers separate the bottom three units. Each unit differs slightly in mineralogy, degree of alteration and elemental enrichment/depletion [47].

#### Sampling

It was our aim to extend the previous sequence (SEK2014) back in time using a continuous sampling approach and Russian coring equipment. The SEK2016 samples were extracted from the lower section of an erosion gully side-wall (Fig 1B). Using a stainless-steel knife, samples approximately 5 x 5 cm with a thickness of 2–3 cm were cut in duplicate (for geochemical and pollen analyses each) from a freshly cleaned surface. A core was then taken from the gully base below the cut surface using a Russian corer (50 cm long, Ø 4.75 cm). Despite best efforts, it was technically impossible to extract a deeper core. No research permits were required for our study site as it is not in protected area or under protected area regulations. Permits are not required unless sampling falls into specific categories such as the extraction archaeological artifacts, extraction of ‘large quantities’ of earth materials, extraction of economically valuable earth materials, removal of animal products or living plants, or where human subjects are involved. None of these apply to our study.

All samples and the retrieved core were sub-sampled at 1 to 1.5 cm resolution in a pressure-controlled laboratory and stored in a cool room at the Department of Geological Sciences, Stockholm University (Sweden). Samples for geochemical analyses were freeze-dried and subsequently milled using a Lab Wizz Type 320 Micro Ball Mill.

### Age dating

Samples for accelerator mass spectrometer (AMS) radiocarbon dating were sent to the Ångström Laboratory (n = 5; Uppsala University, Sweden) and Beta Analytic Inc. (n = 4; Miami, FL, USA; Table 1). Due to a lack of macrofossils, the chronology was based on bulk organic samples. One sample (373 cm) did however contain unidentifiable plant remains, which gave ages within 100 ^14^C years of the corresponding bulk date. The calculation of sedimentation rate and the interpolation of ages for undated samples was conducted using Bacon [48], version 2.2, run through the open-source statistical platform R, and using the SHCal13 calibration curve [49].

**Table 1:** Sample information and AMS radiocarbon dates from the Ångström laboratory (Ua-) and Beta Analytic Inc (Beta-).

*Lab ID*	*Sample Depth (cm)*	*Radiocarbon age (*^*14*^*C age BP ± 1SD)*	*Calibrated Age Range (cal BP)*	*Organic carbon (%)*
**Ua-54862**	315.6	11,632 ± 57	13521–13328	23
**Beta-466037**	338.5	12,110 ± 40	14066–13768	20
**Ua-54863**	359.3	12,304 ± 61	14494–13045	30
**Beta-466034**	372.8	12,360 ± 40	14622–14080	30
Beta-466735*	372.8	12,470 ± 40	14883–14200	30
**Ua-54859**	387.1	13,019 ± 65	15746–15298	15
**Ua-54860**	416.7	13,500 ± 68	16461–15989	12
**Beta-466035**	428.7	13,650 ± 40	16617–16208	7
**Ua-54861**	445.2	14,033 ± 72	17328–16766	6
**Beta-466036**	456.0	12,630 ± 40	15159–14680	1.5

All analyses were made on bulk samples except for Beta-466735*, which was on a plant macrofossil.

### Geochemical analyses

Approximately every other sample was analysed for total organic carbon (TOC), total nitrogen (TN) and stable carbon isotopic composition (δ^13^C) using a Finnigan Delta V advantage flow mass-spectrometer coupled to a CarloErba CN25000 elemental analyser at the Stable Isotope Laboratory (SIL) of the Department of Geological Sciences, Stockholm University (Sweden). All samples were treated with HCl (2M) in an oven at 60°C overnight prior to analysis. The method has a standard deviation of <3% for TOC and TN quantification and 0.15‰ for δ^13^C isotope values.

Approximately every other sample was analysed using a Bruker S8-Tiger WD-XRF analyser equipped with an Rh-anticathode X-ray tube to obtain absolute elemental concentrations at the Department of Ecology and Environmental Sciences, Umeå University (Sweden). 500 mg of freeze-dried and milled sample was placed in Teflon cups sealed with a 2 μm Mylar film [50]. An assessment of the precision across ten replicate pairs showed a maximum relative deviation of <10% for Al, Na, Mg, Si, K, Ti, P, Ca, Mn and Fe, while Sc, Br and Ga showed larger relative deviation (13, 22 and 35%, respectively) but an absolute deviation of less than 2 ppm (2, 2 and 1 ppm, respectively). For Sr (28%), Y (30%) and Zr (38%) the relative deviation was <10% for most replicate pairs, but the maximum relative deviation was elevated due to a single diverging replicate. In terms of accuracy, the relative difference from a certified reference sediment (NCS DC70314; n = 4) was <9% for Al, Na, Si, K, Ti, P, Ca, Mn, Fe, Sc, Rb, Sr, Ga, Br, Y and Zr but slightly higher for Mg (11%). A radiographic image was acquired for the basal core using an Itrax XRF Core Scanner at the Department of Geological Sciences, Stockholm University (Sweden). A step size of 200 μm was applied with a Mo tube set at 55 kV and 40 mA.

All samples were analysed by infrared spectroscopy, using an Agilent Technologies Cary 630 FTIR equipped with an ATR instrument with a single-reflection diamond crystal at the EcoPast Research Group laboratory (GI-1553), Universidade de Santiago de Compostela (Spain). The measurements were performed within the wavelength spectral range of 4000 to 400 cm^-1^. The number of background scans was set to 64 and number of sample scans was set to 150, all with a resolution of 4 cm^-1^. The background was collected between every sample. Samples were analysed in triplicate, with a correlation between the replicates r>0.9.

### Mineralogical and microscopic analyses

Based on the geochemical data, nine samples were analysed for their mineralogy by XRD using a Philips type powder diffractometer fitted with Philips “PW1710” control unit, Vertical Philips “PW1820/00” goniometer and FR590 Enraf Nonius generator at the RIAIDT Facilities at the Universidade de Santiago de Compostela (Spain). The instrument was equipped with a graphite diffracted beam monochromator and Cu radiation source (λ (Kα1) = 1.5406Å), operating at 40 kV and 30 mA. The XRD was scanned from 2° to 65° 2θ using a step size of 0.02° and counting time of 2 s per step.

Ten samples were further selected to determine if the amorphous Si, identified by the geochemical data, was controlled by the presence of diatoms, phytoliths or some inorganic source. Therefore, the silt fraction was extracted from 1–2 g of untreated frozen samples (following [51]). Carbonates were removed with 10% HCl. Organic matter was oxidized using 17% H_2_O_2_ and clay particles were removed after repeated decanting from 100 ml beakers. Sand was removed after 5 s of sedimentation. Residues were mounted in Naphrax® and visually studied under an Olympus CH light microscope using 1000x magnification and immersion oil.

### Pollen analysis

Following the methods used by Faegri et al. [52] fossil pollen grains were isolated from the sediment matrix, extending from the base of the sequence up to 365 cm. After laboratory preparation, a minimum 300 pollen grains were counted per sample at 400x magnification using a Zeiss Primostar light microscope. Identification of pollen grains was made with reference to local and international pollen reference collections. For statistical analysis, taxa that accounted for less than 1% of the pollen record were excluded from the final dataset. The Asteraceae group–where not all individual species accounted for more than 2%–presented in the results is the sum of all individual species identified.

### Statistical handling

A principal component analysis (PCA) based on a direct matrix (i.e., samples as rows, elements as columns) composed of the elemental data including TOC and TN using z-score transformed data in correlation mode with a Varimax rotation was performed with JMP Pro 14 software. These data are compositional data, i.e., they are calculated as relative percentages of a closed sum and thus values of individual variables are affected by changes in the other variables. Z-score transformations were performed to avoid scaling effects and to obtain average-centred distributions [53]. Principal components (PC) were extracted until they explained at least 85% of the variance in the data but also considering the value of interpretation (the relevance for the components for the specific study).

A PCA was similarly performed on the FTIR-ATR measurements but in this case a transposed data matrix was used (i.e., wave numbers in rows, samples in columns). Using this approach, each sample spectrum is decomposed into a number of scores’ spectra accounting for a given proportion of the sample’s spectral variation. This method enables identification of the main constituents of individual samples based on the variation in spectroscopic signal by providing a loading or an “estimated statistical weight” (square of the loading, i.e., partial communality) for a given set of spectral bands. The factor scores enable the identification of the constituents responsible for each PC, where positive/negative scores are higher/lower than average absorbance. Score peaks representing high absorbance within specific spectral bands can then be related to the typical vibration of a chemical component. A combination of co-occurring peaks is in most cases essential. The extraction of components was as above.

In terms of the pollen, all statistical analyses were performed on data that were square root transformed before analysis to down-weight dominant species. Zonation of the pollen sequence was determined using the Constrained Incremental Sum of Squares (CONISS) cluster analysis technique and using the Rioja and Cluster packages in R on the pollen assemblage data mode. All statistical analyses were undertaken using the code-based statistical platform R [54] and stratigraphic plots were produced using C2 [55].

## Results

Sediments were collected between 456 and 315 cm of depth relative to the surface of the sedimentary deposit. A coarse grained, grey sandy layer is found at the base of the sequence (456–450 cm) (Fig 1C). Leading up to 387 cm, the sediment is dominated by silts and clays that are interrupted by sandy layers at 443–440 cm and 415–411 cm. At 387 cm the sediment transitions to an organic-rich brown accumulation. Distinctly paler layers are located at 356–353 cm and 349–345 cm while stark red laminations are found at 330–320 cm. No abrupt boundaries were observed suggesting continuous deposition over the time.

Age modelling reveals that the SEK2016 sequence covers the period from 17,060 to 13,410 cal BP (Fig 1C). Of the nine age dates, one age inversion was found where the bottommost sample at 456 cm returned an age 2000–3000 years younger than expected (Table 1). It is unlikely that the four dates above this sample are all incorrect, particularly given their consistently increasing age with depth. This inversion is potentially explained by contamination of the sample, exacerbated by the very low organic C content (1.5%, Table 1), possibly through root penetration and leaching through the porous grains of the basal sediment. Consequently, we have not included this date in the age model.

In order to facilitate discussion, the profile has been divided into units and sub-units based in the first hand on the CONISS pollen zonation and then the generalized behaviour observed in the other parameters. These include Unit 3 and its sub-units 3c (456–442 cm; 17,060–16,750 cal yr BP), 3b (440–412 cm; 16,710–16,040 cal yr BP) and 3a (410–396 cm; 16,000–15,620 cal yr BP); Unit 2 and its sub-units 2b (395–382 cm; 15,600–15,030 cal yr BP) and 2a (380–364 cm; 14,960–14,420 cal yr BP); and finally Unit 1 and its sub-units 1c (363–355 cm; 14,380–14,210 cal yr BP), 1b (353–339 cm; 14,180–13,910 cal yr BP) and 1a (338–315 cm; 13,890–13,410 cal yr BP). All the original quantitative data can be downloaded from https://bolin.su.se/data/kylander-2021.

### TOC, C/N ratios, δ^13^C and sediment accumulation rates

**Fig 2:**
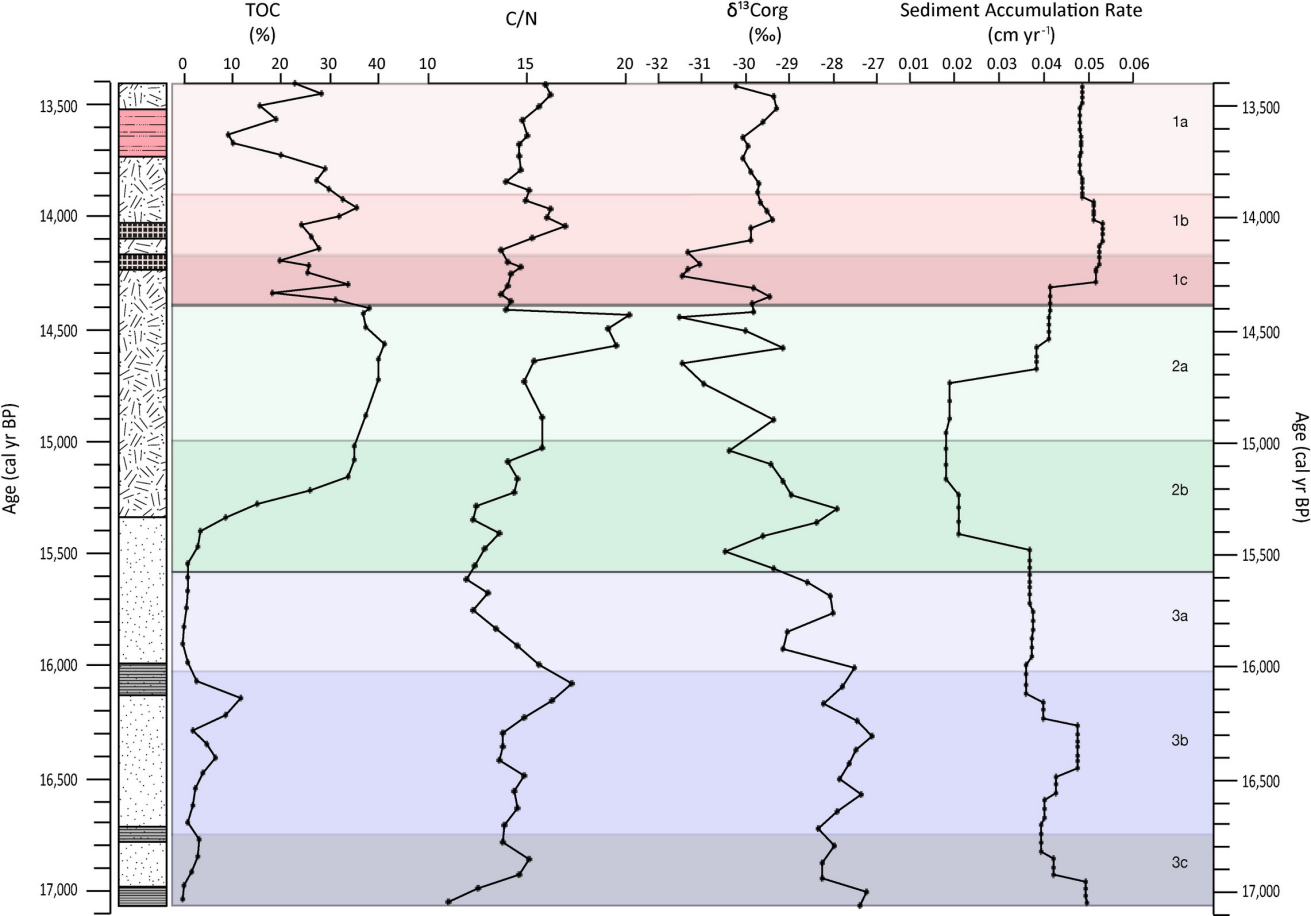
Profiles of TOC, C/N, δ^13^C and sediment accumulation rates by age.

TOC varies significantly throughout the sequence ranging from 0.4% up to 41% (average = 18±28%, n = 59, 2σ) (Fig 2). Unit 3 has the lowest TOC values of the profile averaging around 3%. Within this unit, there is a slight TOC increase in Unit 3c at 16,790 cal yr BP. Unit 3b is noteworthy as it has slightly elevated TOC values, reaching 6.9% and 12% at 16,430 and 16,160 cal yr BP, respectively. TOC is low in Unit 3a but in Unit 2b increases from 1.2% at 15,560 cal yr BP to 35% at 15,100 cal yr BP. TOC remains stable between 35% and 41% in Unit 2a. Unit 1 is characterized by fluctuating TOC values, with peaks observed in most sub-units including Unit 1c (14,310 and 14,150 cal yr BP, 33.7%), Unit 1b (13,970 cal yr BP, 35%) and Unit 1a (13,460 cal yr BP, 28%).

The C/N ratio varies between 11 and 20 throughout the profile, averaging 15±3 (n = 59, 2σ) (Fig 2). Unit 3c at the base of the sequence has the lowest observed values of the profile. Again, Unit 3b is somewhat distinct in having slightly higher C/N values than the rest of the unit, increasing up to 17 (16,080 cal yr BP) before decreasing again to 12 in Unit 3a. Unit 2b has a transitional character in terms of C/N, increasing from 12 to 15 and leading into the maximum values observed in the profile in Unit 2a, peaking at 20 (14,580 to 14,450 cal yr BP). Values drop to 14 in Unit 1c, increase again in Unit 1b to values of 17 (14,050 cal yr BP) only to decrease again to values around 15 in Unit 1a.

The δ^13^C values show a slightly decreasing trend with time, going from -27.4‰ at the base to -30.3‰ at the top of the profile. The most negative values are observed in Units 2a, 1c and 1b where values are as low as -31.5‰ (Fig 2).

Sediment accumulation rates as modelled using Bacon are relatively constant throughout the profile with the exception of Unit 2, where values are up to two to three times lower than in the preceding or following units.

### Elemental geochemistry

The XRF analyses produced a suite of 17 elements with acceptable accuracy and precision (Al, Br, Ca, Fe, Ga, K, Mg, Mn, Na, P, Rb, Sc, Si, Sr, Ti, Y and Zr). In order to reduce the complexity of the data, a PCA was applied to the elemental data including TOC and TN. Four factors explaining 94% of the variance in the data were extracted (Table 2). Principal component 1 (PC1) accounts for 60% of the variance in the data and expresses the behaviour of Na, Mg, Al, Si, Sr, Ca, Sc, Ti, Ga, K and Zr (with positive loadings) versus that of Br, TOC, TN and P (with negative loadings). PC2 explains 13% of the variance and Mn, Fe and P show positive loadings. PC3 and PC4 account for 11% and 10% of the variance, respectively. PC3 is linked to Rb and K variation (positive loadings) versus P (negative loadings) while PC4 represents the behaviour of Y and Zr.

**Table 2:** Variance explained by each principal component (PC) and rotated (varimax) factor loadings.

Variable	PC1	PC2	PC3	PC4
**Variance**	11.4	2.5	2.1	1.9
**Percentage**	59.9	13.0	10.9	9.9
**Cumulative %**	59.9	72.9	83.8	93.6
**Na**	0.97	-0.08	0.18	0.07
**Mg**	0.96	-0.16	0.17	-0.03
**Al**	0.93	-0.24	0.24	0.08
**Si**	0.84	-0.13	0.27	0.32
**Sr**	0.94	-0.07	0.22	0.04
**Ca**	0.94	-0.06	0.11	-0.15
**Sc**	0.89	-0.33	0.19	0.00
**Ti**	0.93	-0.21	0.26	0.03
**Ga**	0.89	-0.16	0.24	0.28
**K**	0.79	-0.20	0.55	0.07
**Rb**	0.37	-0.23	0.88	-0.03
**Y**	0.09	0.15	-0.09	0.94
**Zr**	0.45	0.47	0.33	0.63
**Mn**	-0.08	0.90	-0.06	0.04
**Fe**	-0.13	0.82	-0.30	0.38
**P**	-0.51	0.54	-0.52	0.03
**Br**	-0.89	-0.10	-0.20	-0.29
**%TOC**	-0.92	-0.17	-0.16	-0.26
**%TN**	-0.91	-0.17	-0.17	-0.25

Shaded data are significant at the 0.05 level.

**Fig 3:**
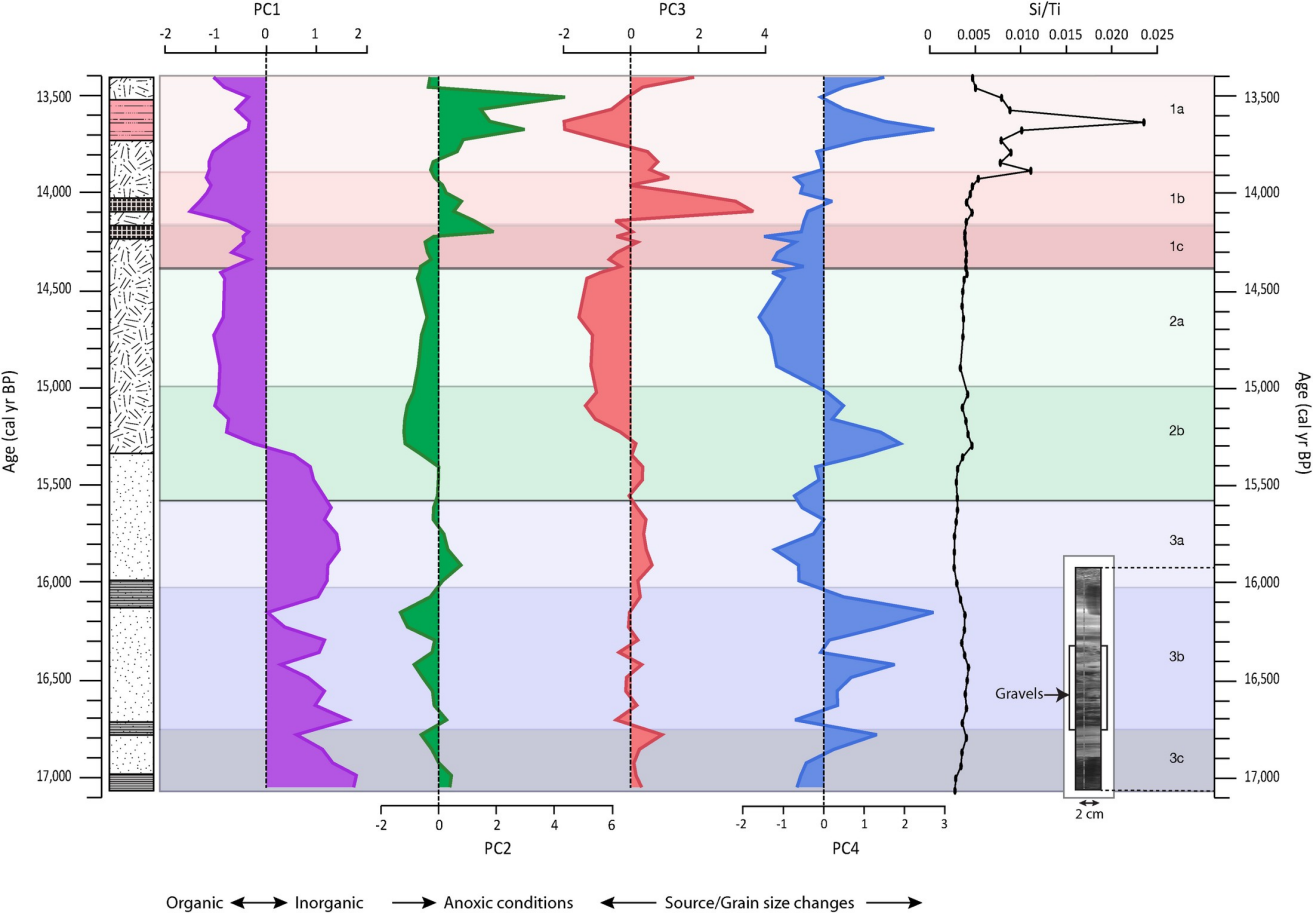
Plots of factor scores by depth of PC1, PC2, PC3 and PC4 as well as Si/Ti ratios by age. The general interpretation of the PC is given. Also included is the available radiograph for the bottommost section which was sampled using a Russian corer, allowing for X-ray scanning.

Change in PCs over time can be plotted using their factor scores (Fig 3). PC1 values are positive in Unit 3 but have a more irregular pattern in Units 3c and 3b in comparison to Unit 3a. Decreases in factor scores occur at 16,790, 16,430 and 16,160 cal yr BP. From the stable period of Unit 3a, values drop to negative scores at 15,560 cal yr BP–that is midway through Unit 2b –and remain stable until they drop slightly in Unit 1c. Factor scores reach the profile minima at 14,100 cal yr BP in Unit 1b, only to become less negative again in Unit 1a. While PC2 factor scores vary around a value of zero for much of the profile, minima are recorded in Unit 3c and 3b at 16,790, 16,430 and 16,160 cal yr BP, while stable, negative factor scores are recorded in much of Unit 2. The most prominent feature of the PC2 profile is the increase at the top boundary of Unit 1c and into Unit 1b, peaking at 14,210 cal yr BP, and the profile maxima in Unit 1a at 13,670 and 13,510 cal yr BP. For the most part, PC3 remains around zero until a period of stable negative values in the upper half of Unit 2b and all of Unit 2a. From this point, PC3 factor scores start to increase, reaching the most positive values in Unit 1b at 14,100 cal yr BP and then the most negative values in Unit 1a at 13,670 and 13,640 cal yr BP. PC4 shows a number of strongly positive peaks in Units 3c (16,790 cal yr BP), 3b (16,430 and 16,160 cal yr BP), Unit 2b (15,290 cal yr BP) and Unit 1a (13,680 cal yr BP). Negative PC4 factor scores occur in Units 3a, 2a, 1c and 1b.

**Fig 4:**
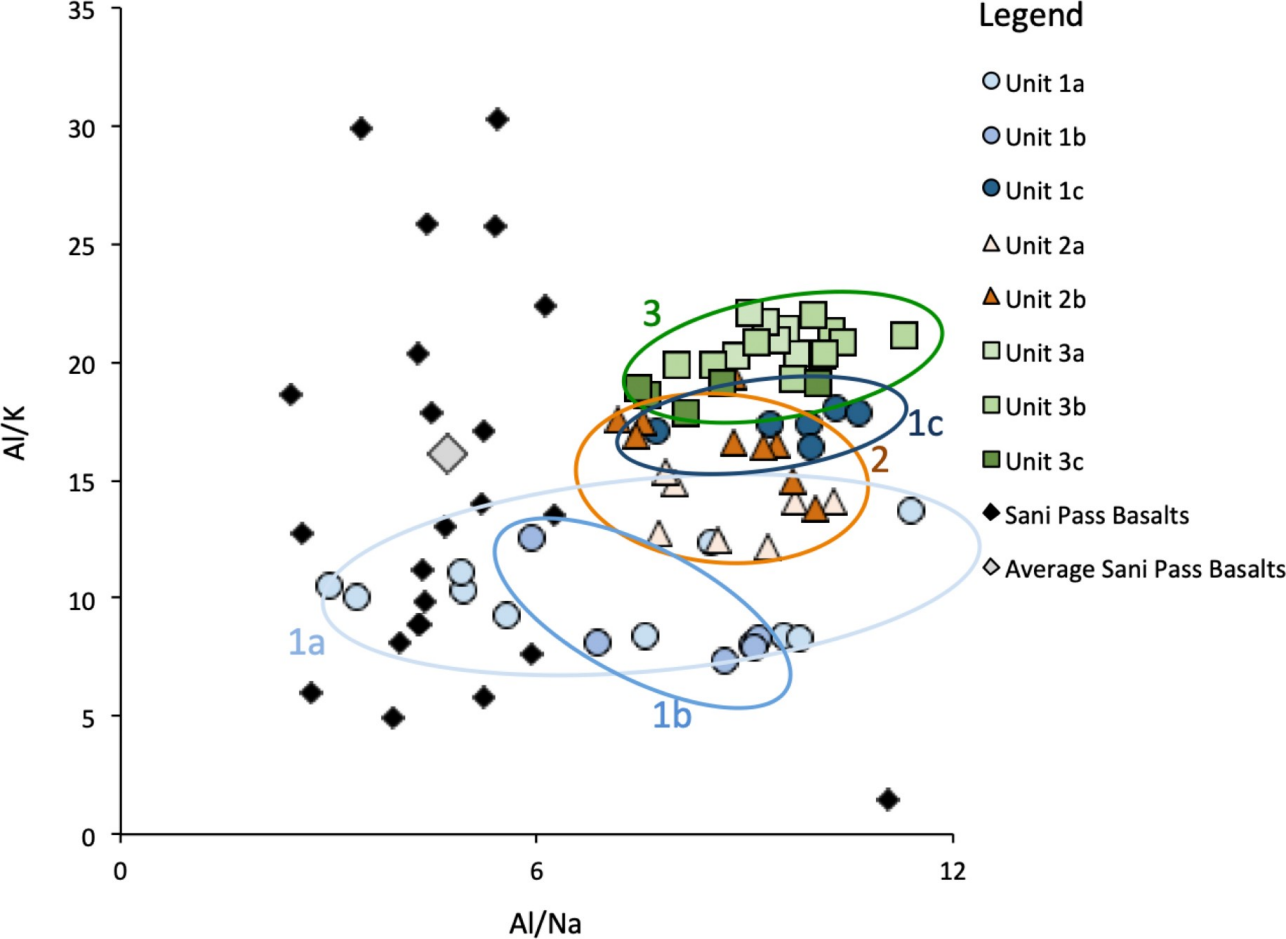
Biplot of Al/K vs. Al/Na by unit for the SEK2016 sequence. Also shown are available measurements of Sani Pass Basalts including an average value.

A biplot of key ratios (Al/K and Al/Na, Fig 4) associated with PC1 was made in order to examine source changes without the effects of organic matter dilution. Samples from Unit 3 plot in a fairly tight field and have the highest elemental ratios of the profile. Samples from Unit 2 and Unit 1c have somewhat lower Al/K ratios and slightly more scatter, but again, show fairly cohesive behaviour. Samples from Unit 1c show quite a narrow range falling between those of Unit 3 and Unit 2. Samples from Units 1b and 1a show lower and far more scatter in elemental ratios (both Al/K and Al/Na) than the rest of the profile.

Samples from the Sekhokong profile were compared to representative local rock samples, the Sani Pass Basalts (individual rocks shown by black diamonds; note that two samples with very high Al/K values are not shown in the plot, and the average of all 25 samples is grey diamond, Fig 4) [47]. Sani Pass Basalts show a wide range of Al/K values (1.5–30.3) while the Al/Na values are more restricted (mostly 2.5–6.0, with an outlier of 11.1). Although the samples from the Sekhokong profile are surely depleted in Na compared to the local rock, the large scatter in Al/K ratios makes it difficult to compare the profile samples with the local rock.

Si/Ti ratios are low in Units 3, 2, 1c and 1b (Fig 3). Values increase at the boundary between Units 1b and 1a, with a double peak occurring in the latter unit. Maximum Si/Ti ratios are observed at 13,860 cal yr BP and 13,640 cal yr BP.

### FTIR-ATR analyses

**Fig 5:**
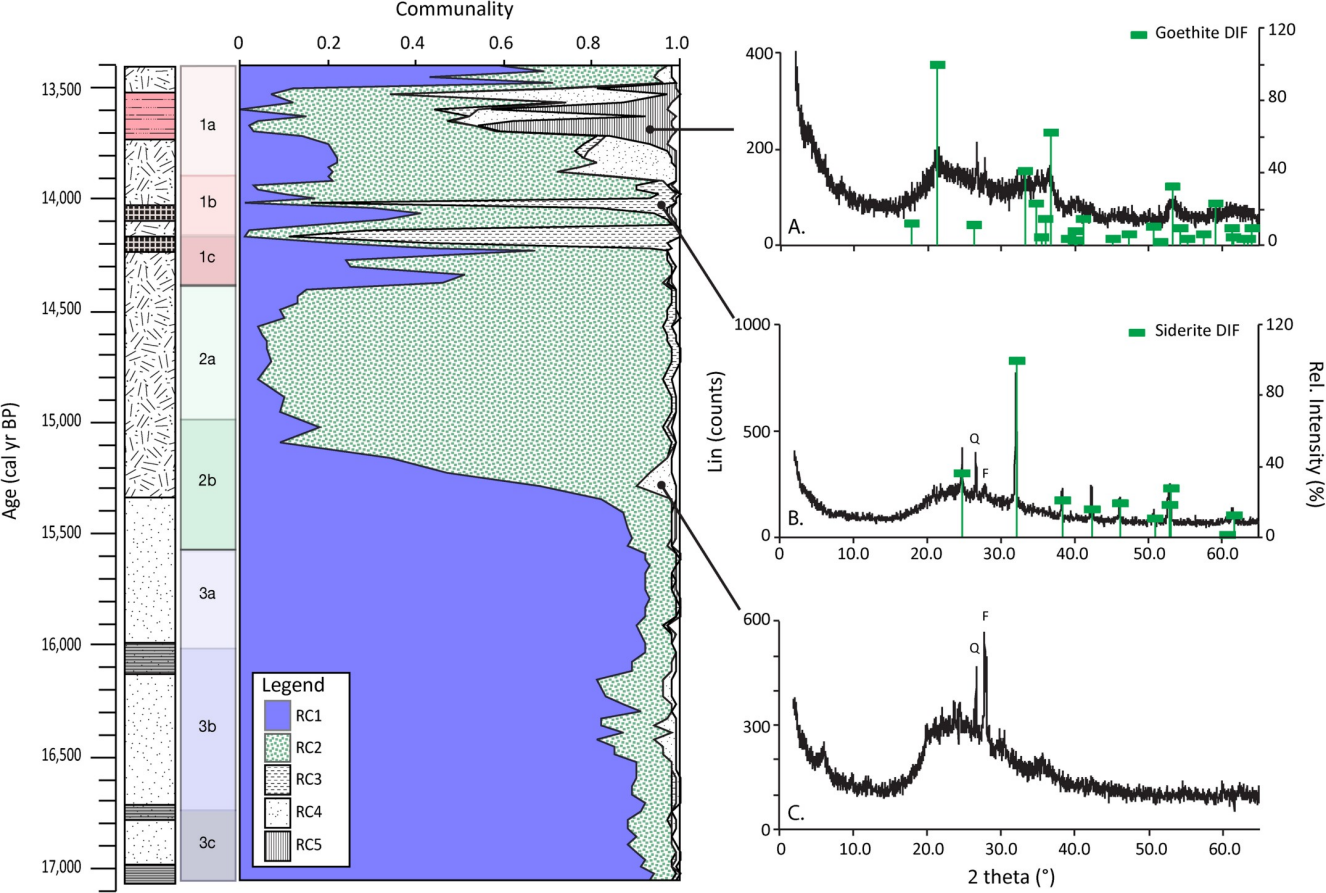
Fractionation of the communalities (i.e., the importance of RC to each sample) over time based on the ATR-FTIR analyses. Representative XRD diffractograms and their sampling location confirm the interpretation of each RC showing typical patterns of (A) goethite, (B) siderite and (C) opal-A.

A PCA was applied to the transposed data matrix of the FTIR-ATR data. This approach was applied, as opposed to that used for the XRF data, because it enables each sample’s spectrum to be separated into basic scores´ spectra, with each accounting for a given proportion (square of the loading of the component, i.e., partial communality) of the spectral variance of the sample. Using the squared loadings of each of the five rotated components (RC), which account for 97% of the data variability, the fractionation of the communalities was plotted (i.e., importance of each component to individual samples) (Fig 5). RC1 explains 52% of the total variance in the data and the squared loadings are highest in Unit 3, explaining on average 89% of the spectral signal in these samples. However, small decreases in Unit 3b at 16,430, 16,335 and 16,200–16,160 cal yr BP are observed. RC1 shows a steep decline in importance in Unit 2b, falling from 0.88 at 15,560 cal yr BP to 0.10 at 15,100 cal yr BP. These low values continue onwards into Unit 2a. Further up the profile, more significant increases in the squared factor loadings of RC1 to 0.50–0.60 occur in Units 1c (14,350 and 14,240 cal yr BP) and the top of Unit 1a (13,490 and 13,440 cal yr BP).

RC2, which explains 37% of the total spectral variance in the data, has a lower weight in Unit 3 (explaining on average 9% of the signal in the samples), with three smaller peaks at 16,430, 16,340 and 16,200–16,160 cal yr BP. RC2 becomes important after a transition in Unit 2b between 15,560 and 15,100 cal yr BP, and then dominates the signal in Unit 2a (0.89). RC2 decreases in importance in Unit 1c (0.49, Fig 5) only to recover somewhat in Unit 1b with peaks at 14,125 and 13,990–13,950 cal yr BP (0.81–0.87), and then decreases again in Unit 1a.

RC3, RC4 and RC5 explain relatively small amounts of the total variance in the dataset (4%, 3% and 2%, respectively), but the expression of these RC is distinct in time (Fig 5). RC3 explains little of the signal observed in samples in Units 3, 2, 1c and 1a (<0.02). However, in Unit 1b RC3 shows peaks with partial communalities of 0.89 and 0.83 at samples dated to 14,210–14,150 and 14,030 cal yr BP, respectively. RC4 shows very weak increases in Unit 3b at 16,430, 16,370 and 16,240–15,160 cal yr BP and slightly stronger signals in Unit 2b (15,290 cal yr BP). The importance of this component increases up to 0.27 (13,910–13,760 cal yr BP), up to 0.40 (13,640 cal yr BP) and, most significantly, up to 0.63 (13,540 cal yr BP) in Unit 1a. Finally, RC5, which remains very low throughout most of the profile, has moderate partial communality (0.36–0.44) in samples early on in Unit 1a (13,710–13,610 cal yr BP), with a weaker peak (0.18) at 13,510 cal yr BP.

The factor scores of these RC are used to identify the spectral signature of the most important components of each individual sample. RC1 has higher factor scores at 3550, 1000, 900 and 520 cm^-1^ (S1 Fig). RC2 has higher score values at 3200, 2919, 2851, 1621 and 1034 cm^-1^. Meanwhile, RC3 shows peaks at 1400, 990, 859 and 738 cm^-1^. RC4 and RC5 show positive peaks at 1100, 792 and 466 cm^-1^ and 3160, 1398, 861 and 440 cm^-1^, respectively.

### Mineralogical and microscopic analyses

Samples were selected for XRD analysis as a way to confirm the interpretation of spectral signals from FTIR-ATR analyses. These included samples associated with increases in RC3 (14,180, 14,150 and 14,030 cal yr BP), RC4 (15,290 and 13,870 cal yr BP) and RC5 (13,680, 13,640, 13,610 and 13,510 cal yr BP)(representative samples shown in Fig 5). Diffractograms of samples associated with RC3 show a pattern that corresponds to the siderite reference [56]. Samples analysed to represent RC4 displayed a broad peak around 20–25° in the XRD diffractogram, which is indicative of amorphous silica. Those samples linked to increases in the importance of RC5 show a diffractogram corresponding to goethite [57].

Ten samples were examined in order to determine the dominant form of amorphous silica. This was a qualitative assessment of the samples but the dominance of diatoms in samples found at deeper depths as opposed to phytoliths in the samples closer to the surface was clear (S2 Fig).

### Pollen

**Fig 6:**
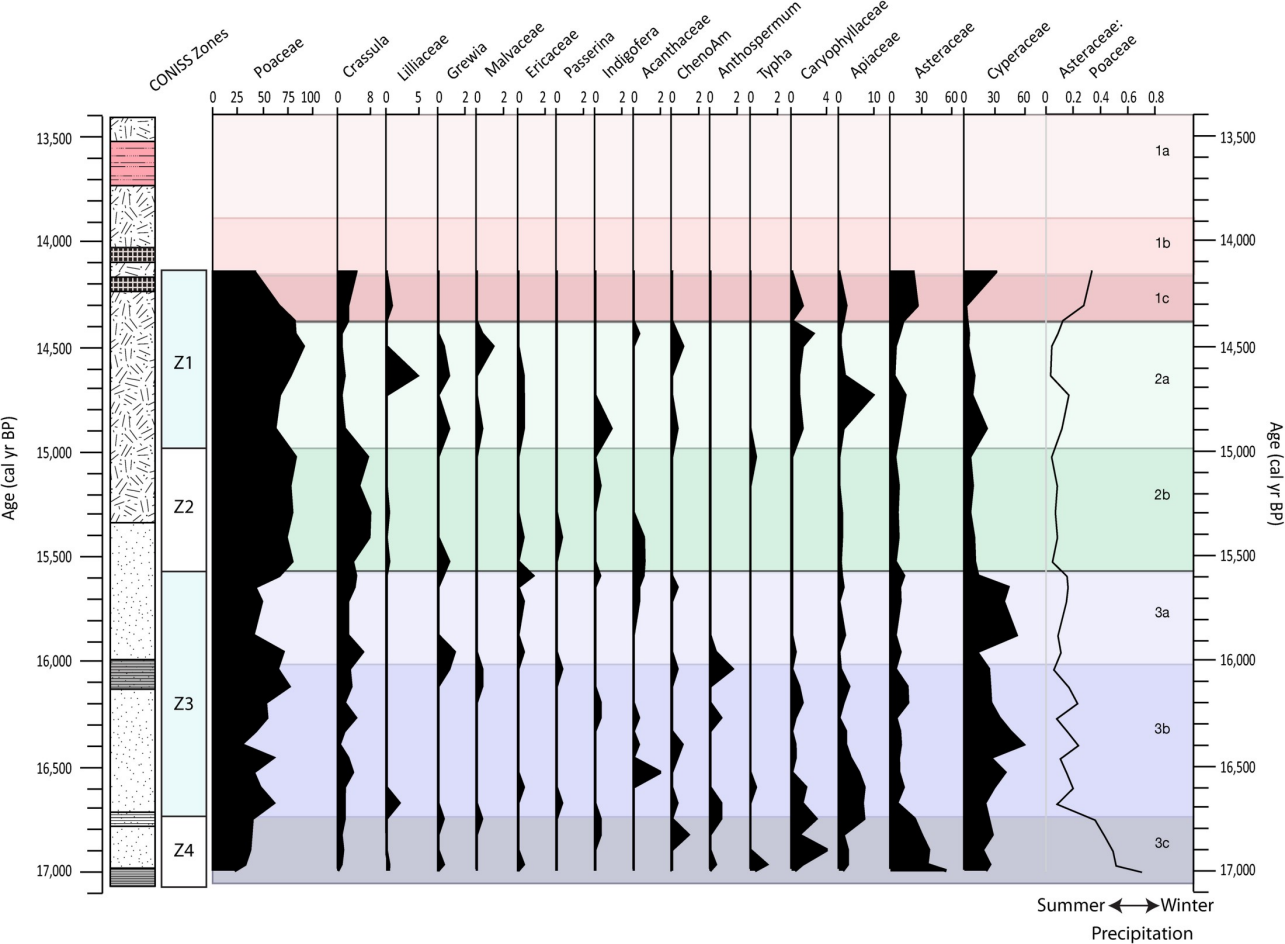
Pollen percentage diagram including the Asteraceae: Poaceae ratio.

CONISS segregated the pollen into four zones, which correspond to Unit 3c; Units 3b + 3a; Unit 2b; and Unit 2a and 1c (Fig 6). The fact that Unit 1c is only represented by two samples likely explains its grouping with Unit 2a. No pollen analyses were made for Units 1b and 1a and in attempts to extend the profile upwards, a pollen data point at 14,140 cal yr BP was included from Fitchett et al. [35]. To interpret environmental and climatic changes, which the pollen sequence represents, pollen counts and ratios of Asteraceae to Poaceae are plotted against age. The sequence is dominated by Poaceae, Cyperaceae and Asteraceae, with Poaceae representing the most dominant species, with a mean percentage composition of 59.8%. In general, in Unit 3c Asteraceae is important, Units 3b and 3a are dominated by Cyperaceae and Unit 2 by Poaceae. The profile also contains small mean percentages of Crassula (2.7%), Apiaceae (2.1%) and Caryophyllaceae (0.7%). Even small mean percentages of a range of wetland species and small shrubs are also noted, namely Liliaceae, *Typha* and Apiaceae.

## Discussion

The four lines of evidence used to reconstruct the paleoenvironmental changes recorded in the SEK2016 sequence include: (i) TOC, C/N and δ^13^C analyses, (ii) XRF elemental data, (iii) FTIR-ATR spectral and XRD analyses and (iv) fossil pollen data. Prior to making our reconstruction, we must first establish what each proxy means at Sekhokong. We reiterate that the majority of the analyses were made on every second sample with the exception of FTIR-ATR, which was made on every sample (Fig 5).

### Proxy interpretation: Geochemical data

TOC is linked to catchment productivity and decomposition rates and increases during periods with high productivity and lower decomposition. C/N and δ^13^C relate to the origin of this organic material in terms of the relative contribution of algal versus terrestrial sources and C_3_ or C_4_ production pathways. The interpretation of the PCA results of the elemental and spectral data is, however, more complex. Each extracted PC can be thought of as representing a single sediment component/process or opposing sediment component/process on the negative and positive side of a PC. The importance of each PC over time can be gauged using factor scores plotted against age, where zero represents the average of this component/process and positive/negative scores represent a relative strengthening/weakening (depending on the sign of the loading) of this component/process (Fig 3). A similar approach can be taken using the squared loadings of each RC plotted against time, which shows the importance of each RC for an individual sample (Fig 5).

The PCA on the elemental data extracted four PC, with PC1 being by far the most important (i.e., accounting for a large proportion of variance) (Table 2). Rocks in the area are dominated by relatively geochemically homogenous basalts and are composed of plagioclase feldspar, olivine, pyroxene (augite, pigeonite) and opaque minerals (<2%, ilmenite, pyrite, titaniferous magnetite, magnetite). Amygdales are found in all units, with the exception of the Sakeng Unit, and are filled with calcite, quartz and zeolites [47]. This combination of minerals readily provides hosts for the rock-forming elements positively associated with PC1 [58]. The negative loading of TOC, TN, P and Br, all elements associated with organic matter, leads us to interpret PC1 as representing the balance between inorganic and organic components in the sediment, with positive/negative values representing more/less inorganic input. PC1 is expressed in the FTIR-ATR data through RC1 and RC2. RC1 shows a spectral signature typical for clay minerals [59] and therefore the input of inorganic material (Table 3, S1 Fig). In contrast, RC2 is interpreted to represent organic matter because it has a spectral signature with peaks recognising OH groups, aliphatic (fats, waxes and lipids), humic compounds, aromatics and polysaccharides [60,61].

**Table 3:** Main peaks used to characterise the RC as extracted by PCA of the FTIR-ATR data, the associated molecular bond and vibration as well as what component these absorption bands can be associated with.

RC	Wavenumber (cm^-1^)	Peak assignment	Component	Reference
**RC1 RC2 RC5**	3700–3000	Stretching and bending vibrations O-H		59, 60, 62
**RC1**	1200–400	Stretching and bending vibration of SiO and Si-O-M (M = Al, Mg, etc)	Clay minerals	59
**RC2**	2919 and 2851	Asymmetric stretching of C-H	Aliphatic	60, 61
	1621	C-O vibration C = O and COO^-^ groups	Humic compounds Aromatics	
	1034	C-O stretching	Polysaccharides	
**RC3**	1400, 859 and 738	Asymmetric vibration, out-of-plane bending vibration and in-plane bending vibration of CO_3_^2-^	Carbonate	59
**RC4**	792	Symmetric stretching vibration of Si-O-Si	Biogenic silica	62
	466	Bending vibration of SiO_4_		
	1100	Asymmetric stretching vibration of SiO_4_		
**RC5**	1398	Fe-OH vibration	Iron hydroxide	63
	861	Bending vibration of the hydroxyl groups of iron-oxide		

PC2 loads Mn, Fe and P, as well as Zr (weakly). There are a number of minerals hosting Mn and Fe in this system and these elements can be transported in a dissolved form, bound to organic matter or as particles [62]. Both Fe and Mn are redox sensitive elements that precipitate under oxic conditions but can be remobilized when anoxic conditions prevail [63]. Phosphorous can co-precipitate with Fe as FePO_4_ in aquatic systems [64]. Increases in PC2 are thus associated with the precipitation of Fe, Mn and P. The identity of the precipitated minerals can be gleaned by examining RC3 and RC5 from the FTIR-ATR data, which increase in importance when PC2 shows high positive factor scores (Figs 3 and 5). RC3 has a spectral signature typical of carbonates [59] (Table 3, S1 Fig). Elemental data show low concentrations of Ca and Mg but relatively high concentrations of Fe for the samples showing high RC3 loadings, indicating that RC3 represents the presence of siderite (iron-carbonate; FeCO_3_) rather than that of calcium carbonate. The presence of siderite in these samples was independently confirmed by XRD analysis (Fig 5). Siderite formation is restricted to alkaline reducing conditions and high CO_2_ partial pressure [65], such as anoxic environments with high microbial oxidation of organic matter [66]. The formation of siderite is often one of the first reactions to occur in the sediment, which makes the presence of siderite minerals a good proxy for reducing conditions in paleoenvironmental studies [67]. RC5 represents variations in goethite (α-FeOOH), the most common Fe oxide in natural environments [68], based on the absorption represented by vibration of OH groups, Fe-OH and hydroxyl groups of iron oxides [69] (Table 3). The presence of goethite was also independently confirmed by XRD analysis (Fig 5). It can be difficult to use goethite as a proxy to reconstruct processes and paleoenvironmental conditions because it is one of the most common Fe-oxyhydroxides in natural environments [68,70]. There are multiple formation pathways, implicating different processes that require different conditions. However, the typical formation setting is under cool and wet conditions, like those found at the high elevations at our study site [68]. It also requires high organic matter content and acidic pH [71]. Lowering of the pH and increased oxygen levels would favour precipitation of goethite over siderite [72]. Goethite is also associated with alteration of siderite and hematite [56,73,74] and may thus be a result of post-diagenetic processes [74,75]. This implies that PC2 reflects changes in redox conditions, but finer compositional details are described by RC3 and RC5.

PC3 and PC4 pair the behaviour of K and Rb and Y and Zr, respectively. Both K and Rb are associated with feldspars and can substitute for one another in the mineral lattice. These elements are both enriched in the Agate Vale Unit, for example, while Rb is enriched in the Mkbomazana Unit [47]. Factor scores for PC3 are near-zero in Unit 3, the lower part of Unit 2b as well as in Unit 1c and these samples plot close to one another in biplot space, indicating they have a similar source (Fig 4). The largest positive anomalies are seen in Units 1b and 1a, which show a distinct elemental signature in comparison to the rest of the profile. While we cannot exclude that the behaviour of PC3 is linked to grain size changes, we note that this PC shows little change at the base of the profile where, as seen in the radiographic image, we have considerable grain size variations (Fig 3, inset). Rather, PC4 increases in the observed layers with less coarse material and decreases in sand and gravel layers, PC4 represents the behaviour of Zr and Y, both hosted in zircon, which is often found in silt-sized fractions [76]. As with PC3, we cannot exclude the effect of source changes, but suggest that grain size is the main control for PC4, with lower/higher values indicating coarser/finer grains.

Based on the main characterising peaks, RC4 is interpreted to signal amorphous Si (Table 3, S1 Fig). Given the sedimentary setting, it is most likely that this is biogenic Si (BSi). This was independently confirmed by the XRD analyses (Fig 5), showing a diffraction pattern typical for opal-A, which is BSi formed as phytoliths from plants or frustules formed by diatoms, radiolarian and sponges [77]. The elevated BSi identified by RC4 is also supported by the microscope observations of diatoms and phytoliths (S2 Fig) and increased Si/Ti values (Fig 3). Variations in BSi abundance over time may be a result of changes in diatom productivity [63,78] with an increase suggesting higher productivity, indicating conditions with a relative increase in humidity [79]. Diatoms only stand for a part of the phytoplankton community however, and these variations may also reflect changes within the community itself [63,78]. Thus, the possible input of phytoliths and other forms of BSi also needs to be taken into account.

In summary, PC1, RC1 and RC2 are indicative of changes in the proportion of inorganic and organic material; PC2, RC3 and RC5 are linked to changes in redox conditions; PC3 and PC4 are affected by changes in sediment source and grain size; and RC4 represents the input of BSi (Table 4).

**Table 4:** Summary of proxy interpretations.

	XRF PC	FTIR RC	Other Data
**Organic/inorganic matter**	PC1	RC1: inorganic matter	TOC
		RC2: organic matter	
**Redox conditions**	PC2	RC3: Siderite	
		RC5: Goethite	
**Sediment source and/or grain size changes**	PC3		Al/K vs. Al/Na
	PC4		
**BSi**		RC4: amorphous Si	Si/Ti

### Proxy interpretation: Pollen

The three most common pollen types identified at all studied wetland sites in eastern Lesotho are Cyperaceae, Poaceae and Asteraceae [35,80–82] (Fig 6)–a suite of very broad cosmopolitan families that reflects the most dominant contemporary and Holocene plants [35,81–85]. Cyperaceae (sedges) is a semi-aquatic plant family, indicative of wet marsh conditions [83,86]. A relative increase in Poaceae pollen reflects a proportional increase in grassland area, and when paired with a decrease in Cyperaceae pollen, locally represents wetland desiccation at least at a seasonal scale [31,83,87]. While Poaceae and Asteraceae on their own may be less climatically informative due to their diverse ecological ranges [88], the ratio between Asteraceae and Poaceae is used to determine the strength of precipitation seasonality. The larger the proportion of Asteraceae pollen relative to Poaceae, the greater the inferred influence winter precipitation [89–91] where scores >0.6 are indicative of distinct winter rainfall conditions, scores <0.2 of summer rainfall conditions, and fluctuations between these representative of proportional changes in winter precipitation amount [92].

The remainder of the fossil pollen sum comprises smaller relative abundances from a range of plant families. Periods of heightened species diversity are noted throughout the profile, comprising increases in the relative abundance of pollen from small, flowering shrubs, which can very broadly be interpreted as indicating discrete periods of warmer temperatures. It is, however, possible that these peaks in species diversity reflect an increase in wind speed, which would expand the radius from which pollen at the site originates [93–95]. An increase in Caryophyllaceae and Apiaceae pollen, by contrast, can be interpreted to reflect cooler periods, as these plant families are notably cold-tolerant [96–98]. Cyperaceae, Apiaceae and *Typha* are semi-aquatic families, and an increase in their percentage composition reflects an increase in moisture [96,99]. Furthermore, as aquatic species, fluctuations in their relative pollen abundance are more indicative of the plant communities within the wetland itself, rather than the broader alpine region [31]. Cheno-Am (the group of morphologically indistinguishable Chenopodiaceae and Amaranthaceae), Acanthaceae and *Crassula* by contrast, reflect dry conditions [88,90,96,100]. In particular, Cheno-Am reflects an increase in evaporation, rather than, necessarily, a decrease in precipitation [31,102]. Considering seasonality, the increased presence of Fynbos species, albeit comprising small proportions within the high Maluti Mountains including Ericaceae, *Anthospermum* and *Passerina*, may indicate shifts to colder and wetter conditions, as these species are best suited to the Cape Floristic Kingdom in the WRZ [25,31,101,102]. Notably, these do not appear in significant proportions in the SEK2016 sequence.

### Proxy synthesis: Paleohydrology and paleovegetation

**Fig 7:**
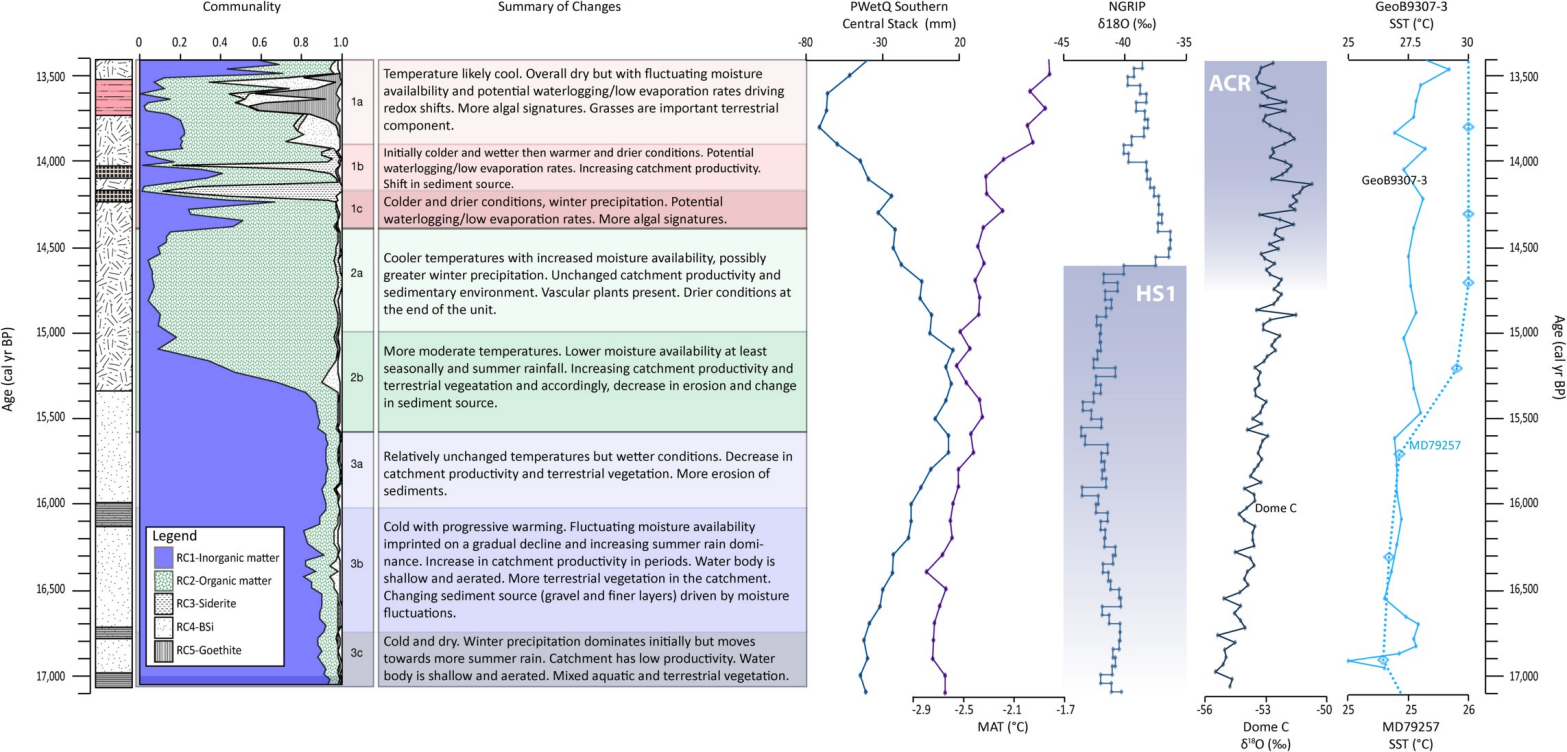
ATR-FTIR analyses were made every sample and as such the fractionation of the communalities is shown here along with a summary of changes in the SEK2016 sequence. Note that changes are described in relation to the preceding unit. We compare with the PWetQ reconstruction for the southern central SRZ and MAT from Chevalier and Chase [103] to provide regional context. In order to track changes in the North Atlantic and Antarctica, NGRIP and Dome C ice core δ^18^O are plotted [104,105]. Also shown are reconstructed SST from two Indian Ocean marine cores, GeoB9307-3 and MD79257, from off the coast of Mozambique near the southern boundary of the present day seasonal ITCZ migration [15,16].

Based on the pollen zones and the general behaviour of the proxy suite, the sequence has been divided into three main units. Broadly, Unit 3 (17,060–15,620 cal yr BP) represents a period dominated by inorganic matter input, which would generally indicate lower productivity and/or preservation under drier and/or colder conditions in comparison to Unit 2 (15,600–14,420 cal yr BP) (Figs 2, 3 and 5). Unit 2 is characterized by higher organic matter content and a slowdown in sediment accumulation rates, suggesting a period with increased vegetation and catchment productivity, under comparatively warmer and sometimes wetter conditions. Finally, Unit 1 (14,380–13,410 cal yr BP) shows contrasting geochemical environments and presents periods of anoxia, potentially driven by water-logging/low evaporation rates during wet winters, and generally drier conditions (see Fig 7 for summary). Since several proxies represent similar compounds or processes, for simplicity in the discussion that follows we refer to inorganic matter (representing low TOC, high PC1 factors scores and RC1), organic matter (higher TOC, low PC1 factors scores and RC2) and BSi (Si/Ti and RC4) (Table 4).

#### Unit 3: 17,060–15,620 cal yr BP (456–396 cm)

Unit 3c (17,060–16,750 cal yr BP 455–441 cm) is dominated by inorganic matter (TOC <3.6%), which suggests relatively low productivity and/or low preservation due to dryer and/or colder conditions (Fig 2). Organic content increases slightly moving upwards to 16,790 cal yr BP and C/N values increase (11 to 15). In combination with the negative δ^13^C values (-28‰), this indicates that the organic matter is a mixture of lacustrine algae and terrestrial C_3_ plants, similar to that found in the area today [106]. PC2 and PC3 show a weak shift mid-way through Unit 3c, which suggests aerated water and a change in sediment source, as supported by the slight separation of Unit 3c samples on the Al/Na axis (Figs 3 and 4). A stronger shift towards positive values is observed in PC4, which together with the radiograph, indicates an upwards fining of the sediment. Variations in the source and grain size of the deposited sediments is ultimately driven by catchment hydrology through changes in for example, the amount or seasonality of precipitation or surface water runoff as controlled by vegetation cover. Pollen indicates the presence of Asteraceae, Caryophyllaceae and Apiaceae, all tolerant of cold temperatures and restricted moisture availability (Fig 6). Unit 3c shows the highest proportion of Asteraceae pollen in the profile, coinciding with a relatively low proportional representation of Poaceae, and peaks in Fynbos species *Anthospermum* and the Ericaceae family, which is interpreted to be a result of an increased proportion of colder season precipitation. Asteraceae then declines sharply, compensated by the cold-tolerant families of Caryophyllaceae and Apiaceae. At the same time, the relative abundance of Poaceae remains largely constant, suggesting a gradual shift toward more dominant summer precipitation at the site. A second peak in Caryophyllaceae and Apiaceae occurs at the end of Unit 3c, indicating a second distinct cool period, which matches with the source change/grain size shift seen in the elemental data. These proxies, in combination, are indicative of a low productivity aquatic system surrounded by a grassy habitat with cold temperatures and dominating winter rainfall, gradually giving way to more summer rainfall over time.

The main feature of Unit 3b (16,710–16,040 cal yr BP; 440–411 cm) is the triple decreases/increases in inorganic/organic matter (Fig 5). While C/N values increase at the end of the unit, δ^13^C values remain relatively stable (Fig 2). The observed shifts in inorganic/organic matter are matched by decreases in PC2, increases in PC4 and weak peaks in BSi, linked to the presence of diatoms (Figs 3 and 5). These changes evoke small increases in catchment productivity of what was likely an aerated, and therefore, shallow water body (or bodies). Vegetation increase in the catchment may explain the shift towards finer grain sizes suggested by positive PC4 factor scores, which is indicative of calmer conditions within the catchment. Variation in the proportional representation of Poaceae, *Crassula* and Cyperaceae likely reflects alternating levels of moisture availability, resulting in fluctuations in the wetland extent during this period. The percentage composition of Cyperaceae generally decreases throughout this period, concurrent with an increase in Poaceae and *Crassula*, and greater proportions of Acanthaceae and ChenoAm, suggesting a gradual reduction in moisture availability at the site. The decrease in Asteraceae and replacement by *Crassula* may infer a shift from winter rain towards more summer rain dominance [91,92]. The percentage composition of both Apiaceae and Caryophyllaceae declines throughout this period, indicating progressive warming at the site. Taking this evidence together, the deposit at this time could have been covered by a dynamic patchwork of wetland vegetation and shallow pools not uncommon in the area at present.

The top sub-unit Unit 3a (16,000–15,620 cal yr BP; 410–396 cm) sees a return to higher inorganic contents and lower organic matter contents (Figs 2 and 5). Both C/N and δ^13^C signal the increased role of algal contributions relative to terrestrial vegetation over time [107] and weakly positive PC2 factor scores evoke the existence of wetter conditions and possible anoxic conditions (Fig 3). These wetter conditions, through more intense rainfall or snowmelt, would enable the physical movement of the coarser material visible in the radiograph and the sandier material observed at the base of this sub-unit and indicated by lower PC4 values. This could also be driven by a decrease in terrestrial vegetation and the loss of soil stabilizing roots [108]. In line with the wetter and more erosive environment indicated by the geochemical proxies, Cyperaceae return with dominance in this unit and signals an increase in wetland coverage (Fig 6).

#### Unit 2: 15,600–14,420 cal yr BP (395–364 cm)

Unit 2b (15,600–15,030 cal yr BP; 395–382 cm) is characterised by a TOC increase from 1.2% at 15,560 cal yr BP up to 35% at 15,100 cal yr BP (Fig 2). This in itself would indicate conditions more favourable to organic matter production and preservation. C/N values increase and δ^13^C become less negative, suggesting a mixture of lacustrine algae and terrestrial C_3_ plants, with an increasing influence of terrestrial organic matter input with time. Sedimentation rates drop sharply over the inorganic-to-organic transition. PC2, PC3 and PC4 cross zero simultaneously (although not all in the same direction) midway through Unit 2b at 15,620 cal yr BP (Fig 3) and samples from this period start to move away from the base of the profile in biplot space (Fig 4). A peak in preserved diatom derived BSi is also observed over this transition (Fig 5). Taken together, vegetation cover and catchment productivity undoubtedly increased during this time, altering the source and grain size of the mineral material deposited in the basin. The pollen is marked by a very high abundance of Poaceae and *Crassula* and low percentages of semi-aquatic Cyperaceae and Apiaceae; this indicates very low moisture availability, at least seasonally (Fig 6). Reduced proportions of Asteraceae:Poaceae pollen would suggest that the majority of precipitation occurred during the summer months, despite the continued presence of small percentages of Fynbos pollen. The very low percentage composition of Apiaceae, and the absence of Caryophyllaceae may indicate more moderate temperatures during this period. It would seem that the precipitation regime changed rather quickly to a distinct summer rainfall regime and grassland coverage expanded.

Unit 2a (14,960–14,420 cal yr BP; 380–364 cm) is the most stable part of the record, with continued higher organic matter content (TOC between 37–41%) (Fig 2) while C/N values peak and δ^13^C values indicate the presence of vascular plants. It would appear that the sedimentary environment remained relatively unchanged from that in Unit 2b, with the exception of perhaps somewhat coarser materials being deposited at this time (Figs 3 and 5). In this unit there is an initial increase in Cyperaceae, Apiaceae and Asteraceae with a concomitant decline in Poaceae and *Crassula*, which suggests an increase in moisture availability, with likely year-round precipitation (Fig 6). The peak in Asteraceae may reflect a return to greater proportions of winter rainfall. Additionally, the eastern Lesotho highlands are home to a number of cold-adapted Asteraceae species, including *Helichrysum*, which may comprise this peak in Asteraceae (Carbutt and Edwards, 2004). Caryophyllaceae and Apiaceae also increase, suggesting colder conditions. The peak in Cyperaceae and Apiaceae pollen is followed by a peak in semi-aquatic Lillaceae pollen. This may indicate the prolonged presence of moisture allowing for standing surface water, preferred by endemic species such as the Sehlabathebe Lily [85] and the re-emergence of a wetland habitat. At the end of Unit 2a, semi-aquatic species become less prominent and Poaceae and ChenoAm percentages increase again, starting a shift in the balance towards grasslands habitats again.

#### Unit 1: 14,380–13,410 cal yr BP (363–315 cm)

Unit 1c (14,380–14,210 cal yr BP; 363–355 cm) is characterized by an increase in the proportion of inorganic material (TOC range: 18–31%) along with lower C/N ratios and less negative δ^13^C (Fig 2), indicating an initial move towards more algal signatures and less terrestrial vegetation. PC2 peaks at the boundary of Units 1c and 1b (14,210 to 14,150 cal yr BP) and is matched by a peak in RC3 indicating siderite formation and anoxic conditions (Figs 3 and 5). The chemical signature of the sediments deposited at this time shows a mixing between signatures for Units 3 and 2b (biplots and near-zero values for PC3). The grain size also appears to be relatively unchanged from Unit 2 (PC4). This indicates similar hydrological organisation within the catchment during these periods. Most significantly from the pollen data is that Asteraceae increases, along with smaller increases in Caryophyllaceae and Apiaceae, suggesting cold and dry conditions. The increased Asteraceae:Poaceae ratio evokes a shift towards more cold season-dominated precipitation. Anoxic conditions observed in this unit might be a product of water-logging, low evaporation rates and/or increased microbial activity in the presence of higher amounts of organic matter (Fig 5). Low evaporation rates can be a result of the dominance of precipitation during the cold winter months.

In Unit 1b (14,180–13,910 cal yr BP; 353–339 cm), after a pulse of inorganic material, organic matter contents increase over time, peaking at 14,000 cal yr BP, along with both C/N values and δ^13^C, signalling an increase in productivity linked to greater presence of terrestrial plants (Fig 2). Although PC2 values are near zero at this time, a peak in siderite (RC3) is observed midway through the unit (Figs 3 and 5). PC3 peaks in parallel with the inorganic material pulse (Fig 3). Samples from Unit 1b plot away from sources similar to the profile base, signalling a significant shift in sediment source, and therefore hydrology (Fig 4). Based on these unusual signals midway through the unit, the geochemical data point to initially colder/wetter, and possibly lower evaporation rates and water-logged conditions. This is gradually replaced by relatively drier and warmer conditions with a more productive catchment.

Unit 1a (13,890–13,410 cal yr BP, 338–315 cm) is singular in that it shows maxima/minima for several geochemical proxies and the first significant presence of BSi and goethite (Fig 5). Accordingly, organic matter contents decrease (Fig 2). Interestingly, after an initial C/N decrease at the start of the unit, C/N and δ^13^C remain fairly stable suggesting little change in the organic matter source over time (Fig 2). Meanwhile, PC2 reaches profile maxima, which coincide with peaks in goethite (Figs 3 and 5). Alternating layers of siderite and goethite can coexist as a consequence of fluctuations in the water table and access to organic matter for degradation. Goethite requires colder conditions, higher redox potentials and more acidic conditions than siderite, which speaks for decomposition of organic matter in drier conditions [69]. PC3 shows its most negative values half way through Unit 1a, and in biplot space, the elemental signatures of the sediment show a large scatter with lowest Al/Na ratios of the record. The overlap of samples from Unit 1a with signatures typical of Sani Pass Basalts, suggests the input of relatively unweathered material. PC4 has a positive peak with a similar magnitude to that seen in Unit 3b, indicating fine-grained material input and reduced hydrological intensity. The increasing dominance of BSi at 13,905–13,760 and around 13,640 and 13,540 cal yr BP, is apparent in both the Si/Ti and RC4 profiles (Figs 3 and 5). Qualitative microscope observations found that this BSi is associated with low diatom contents and high abundance of phytoliths with a parallelepiped elongated long morphology, specific for grasses (Poaceae), but non diagnostic for the subfamilies [109–111]. While we have no pollen analyses at these depths, previous pollen studies at Sekhokong (SEK2014) show a peak in grass species (Poaceae) at this time [35]. Given this evidence, we interpret this to be a dry, grassland-dominated period with likely cooler conditions supporting the formation of goethite and increased inorganic matter input at the top of the profile.

### Regional context and implications

Paleoenvironmental changes reflected in the SEK2016 sequence, for the period 17,060 to 13,400 cal yr BP, are demonstrably complex. However, these should be considered in the context of the site locality, in this case a north-exposed (i.e., sun-exposed) slope in a high alpine environment, and in one of the coldest and among the wettest regions of semi-arid southern Africa [103]. Moraines dated to between ~19,350 and 14,700 cal yr BP on the adjacent south-facing aspect to our site indicate that this was a cold period in the high Drakensberg [20]. However, the work by Mills et al. [20] also highlights strong micro-climatic and geomorphological contrasts between north- and south-facing slope aspects along the Sekhokong Range. With our site being on the much warmer north-facing aspect, it would have prevented permanent snow and ice accumulation, and rather yielded continuous slow sedimentation through on-going slope processes. Given that wetland species are present throughout the SEK2016 record, conditions must have been relatively wet. This is the climatic “umbrella” under which we consider the observed *relative* changes.

On millennial timescales, colder temperatures in southern Africa are often linked to drier conditions [9,23,33,103,112] and increased proportion of winter precipitation [113]. Nonetheless, considerable climatic complexity has been observed in the southern-central portion of the SRZ during the Pleistocene-Holocene transition; this has been attributed to the variable influence of both tropical and temperate systems over time [9,103]. Regional changes are represented here by pollen-based reconstructions of precipitation during the wettest quarter (PWetQ)(southern-central precipitation stack) and mean annual temperature (MAT) for the SRZ [103] (Fig 7). Changes in precipitation at Sekhokong are broadly parallel with the southern-central stack showing generally drier (~Unit 3), wetter (~Unit 2) and driest (~Unit 1) conditions at the start, middle and end of the studied time interval, respectively. There is a shared trend between SEK2016 and the MAT stack towards increasingly warm conditions over time. However, Sekhokong deviates from these overarching trends, showing more nuanced decadal to centennial scale variability where both colder and warmer periods can be wetter or drier. These offsets are likely due to a combination of (i) climatic factors such as changes in rainfall seasonality and evaporation rates; (ii) orographic effects where blocking of moisture coming from the Indian Ocean by the escarpment has previously been suggested as a source of signal lag at Sekhokong [24,35]; and/or (iii) the differing location of individual sites along oceanic-terrestrial temperature and moisture gradients [103]. The fluctuations inferred by the Sekhokong geochemical data strengthen previous interpretations based on stable isotope data from archaeological sites in Lesotho, which suggest an unstable regional climate during the Pleistocene-Holocene transition [43].

The oldest part of our sequence (17,060–16,750 cal yr BP, Unit 3c) indicates cold conditions with increased precipitation likely during autumn-winter-spring. This is in strong agreement with an increasing body of recent literature that has supported relatively humid conditions over southernmost regions of southern Africa, which included an expanded WRZ in northerly and easterly extensions during the late LGM [6–11]. Not only were SWW (and the associated passage of cold fronts) more intense and/or shifted in an equatorward direction during this time over southern Africa [e.g., 9], but responded in a similar manner over South America [114] and Australia [115].

The Sekhokong sequence shows increasingly warm conditions, promoting the accumulation and/or preservation of organic matter, starting from 15,600 cal yr BP (Unit 2b) (Fig 7). An organic matter maximum during wetter and cooler conditions occurs 14,960 to 14,420 cal yr BP (Unit 2a), which coincides with wetland establishment at several sites in southern African [116,117]. This ecosystem shift overlaps in time with the coldest part of the HS1 in the Northern Hemisphere (16.1 to 14.7 ka) [118], which on longer timescales is linked to migration of the ITCZ [101,103]. During HS1, there was also a slowdown in the Atlantic Meridional Overturning Circulation (AMOC), with warming of oceans surrounding southern Africa [119,120], including in the Indian Ocean as shown in marine cores GeoB9307-3 and MD79-257 from off the coast of Mozambique [15,16]. These warm oceans led to wetter conditions, not only in the WRZ but also in the SRZ [9,103]. This may have been driven by increased synoptic effects between temperate and tropical systems, generating temperate-tropical troughs (TTTs), which cause heavy rainfall events over parts of southern Africa [121].

The ACR, which interrupted the last deglaciation seems manifested through a return to colder, drier conditions with cold season precipitation seemingly strengthened again at Sekhokong, most markedly starting at 14,380 cal yr BP (Unit 1c). In general, Unit 1 presents cold and dry conditions as expected for the ACR in the SRZ. During the ACR, westerly storm tracks shifted northwards with the expansion of Antarctic ice. However, this also had the effect of pushing the Subtropical Front northwards, reducing the inflow of moisture to the SRZ [119] and thus accounting for overall drier conditions. Unit 1 is special in terms of the variable redox conditions it presents. We suggest that these conditions are created during the break down of existing organic matter under dry conditions and/or water logging. The latter would be created when the majority of precipitation falls during the colder winter months. We also note that for the small time slice between 14,180 and 13,910 cal yr BP (Unit 1b) conditions do not follow the general “cold and dry” ACR trend, becoming first colder and wetter and then warmer and drier. This is perhaps not unexpected given that we are siting in a sensitive, high-alpine environment where there is the additional complexity of orographic effects, but also in that the ACR itself has its own internal structure as seen in Antarctic δ^18^O records [122 and references therein].

## Conclusions

Here, we have presented the first relatively high temporal resolution paleorecord of sedimentary, chemical, biological and climatic evolution for a site in the high Drakensberg of southern Africa during the Late Glacial (17,060–13,400 cal yr BP). While SEK2016 broadly follows regional changes in precipitation and temperature, finer decadal and centennial variability is revealed. Early in our record, the data support a relatively humid environment with considerable cold season precipitation. There is a significant flip in the system that occurs 15,600 to 15,030 cal yr BP that sees warmer conditions and a shift to summer dominated rainfall. Starting 14,380 and continuing to the top of our record, cold and dry conditions occur with winter precipitation potentially causing waterlogging and anoxic conditions in the sediment. Although there is some variability in climatic conditions during this timeframe, we interpret this interval to be a response to the ACR. Hence, the Late Glacial period was one marked by considerable climatic fluctuation and bi-directional environmental change; something not identified in previous studies for this region. We thus caution studies making inferences that the Late Glacial period in southern Africa as a whole, spanning several thousand years, represented *either* ‘wetter’ *or* ‘drier’ conditions, as it would seem that both moisture and thermal conditions shifted and fluctuated during this time.

## Supporting information

S1 Fig Factors scores for the individual RC used to identify the most important components of each sample. (TIF)

S2 Fig Examples of microscopic images examining BSi contents with a 40x magnification using an Olympus CH light microscope. The selected images represent the general content of the samples. Image A and B present samples from 13,640 and 13,870 cal yr BP, respectively, which are characterized by low diatom content and high abundance of long elongated phytoliths. Image C and D present samples from 15,170 and 16,430 cal yr BP, respectively, and have high diatom content.
(TIF)
