# Supplementary figures and images for: Late glacial (17,060–13,400 cal yr BP) sedimentary and paleoenvironmental evolution of the Sekhokong Range (Drakensberg), southern Africa
Source: PLoS One. 2021 Mar 17;16(3):e0246821. doi: 10.1371/journal.pone.0246821 (PMC7968709; doi:10.1371/journal.pone.0246821)

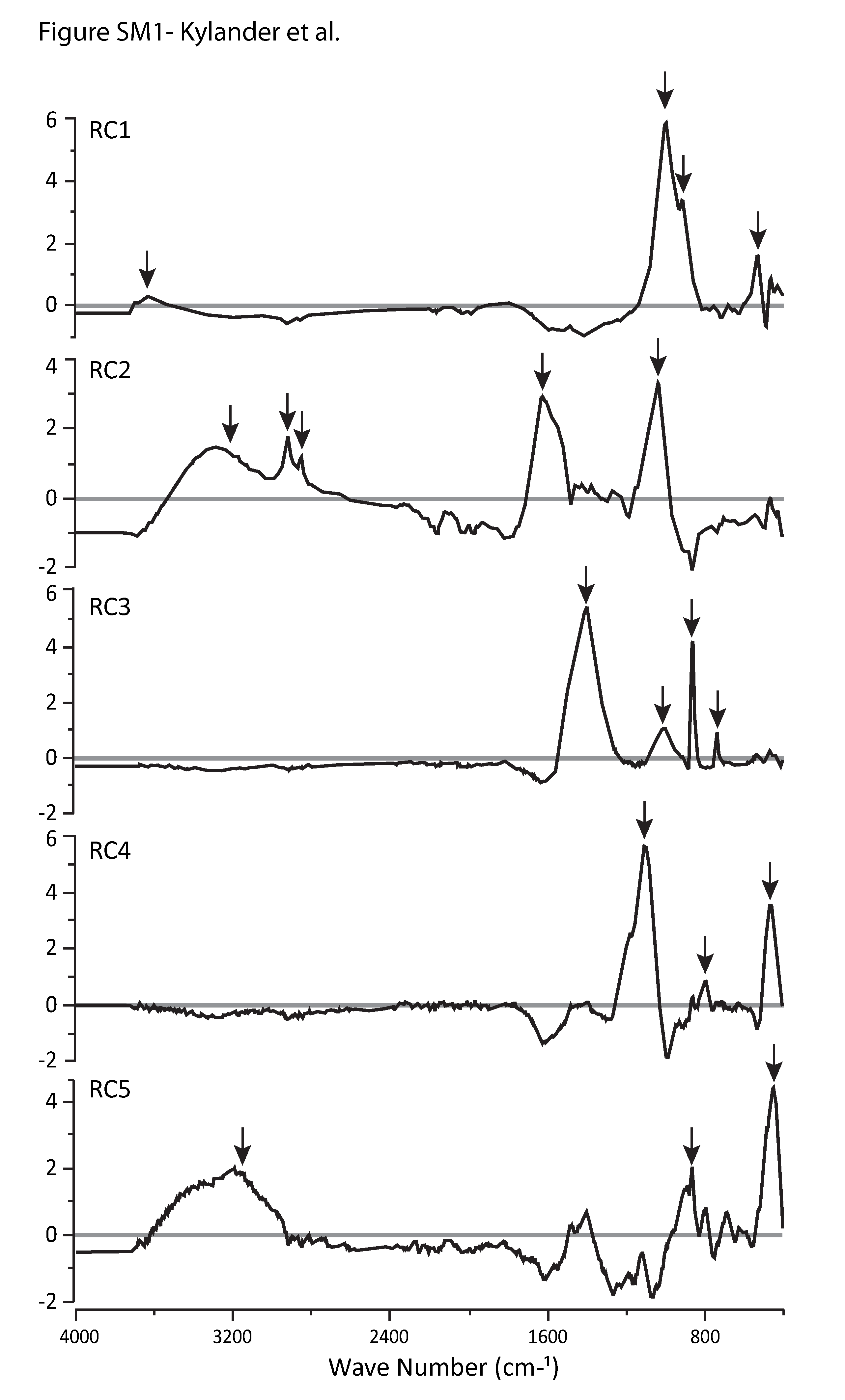

Supplement: S1 Fig — (TIF) [file pone.0246821.s001.tif]

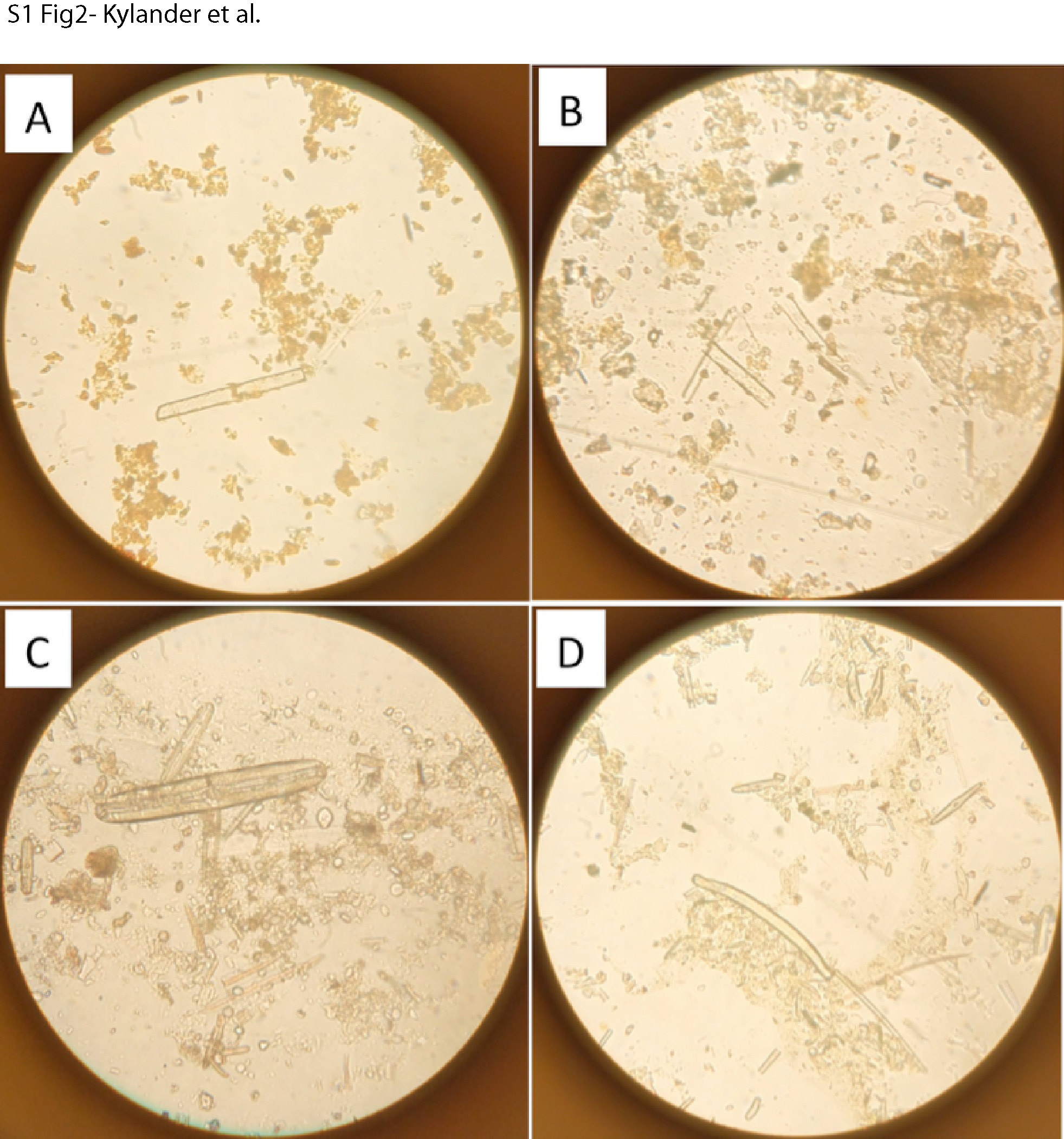

Supplement: S2 Fig — The selected images represent the general content of the samples. Image A and B present samples from 13,640 and 13,870 cal yr BP, respectively, which are characterized by low diatom content and high abundance of long elongated phytoliths. Image C and D present samples from 15,170 and 16,430 cal yr BP, respectively, and have high diatom content. (TIF) [file pone.0246821.s002.tif]
